# 2024 Argentine Group for Extracellular Vesicles (GAVE) Workshop: promoting science in challenging times

**DOI:** 10.1242/bio.062002

**Published:** 2025-06-10

**Authors:** Gonzalo Germán Guendulain, Ana Lis Moyano, Vanesa Mattera, Melisa Carolina Monteleone

**Affiliations:** ^1^ Centro de Investigación en Medicina Traslacional “Severo R. Amuchástegui” (CIMETSA), Instituto Universitario de Ciencias Biomédicas de Córdoba (IUCBC), Consejo Nacional de Investigaciones Científicas y Técnicas (CONICET), Av. Naciones Unidas 420, Barrio Parque Vélez Sarsfield, X5016KEJ Córdoba, Argentina; ^2^ Instituto de Química y Fisicoquímica Biológica (IQUIFIB), Universidad de Buenos Aires, Departamento de Química Biológica, Facultad de Farmacia y Bioquímica, Consejo Nacional de Investigaciones Científicas y Técnicas (CONICET), Ciudad Autónoma de Buenos Aires (CABA), C1113AAD Buenos Aires, Argentina; ^3^ Instituto de Investigaciones Biotecnológicas, Universidad Nacional de San Martín (UNSAM), Consejo Nacional de Investigaciones Científicas y Técnicas (CONICET), 1650 San Martín, Argentina; ^4^ Escuela de Bio y Nanotecnologías (EByN), Universidad Nacional de San Martín, 1650 San Martín, Argentina

**Keywords:** Extracellular vesicles, Argentina, GAVE, Workshop

## Abstract

The Argentine Group for Extracellular Vesicles (GAVE) was established in 2022 with the objective of bringing together researchers working in Argentina dedicated to extracellular vesicle (EV) studies. Following its successful inaugural meeting in 2023, the II GAVE Workshop was held on 12-13 September 2024, at the University of Buenos Aires, Argentina. This event brought together over 140 participants from diverse disciplines, fostering collaboration and strengthening the national EV research field. Moreover, international speakers and renowned experts in their fields shared valuable insights and experiences with the audience. Despite the challenges posed by the national government's funding cuts, the 2024 GAVE workshop showcased the Argentine scientists' strong commitment to high-quality research and the growth of local science in the field of EVs. Supported by international organizations and local companies, the II GAVE Workshop prioritized inclusivity and provided valuable networking opportunities, particularly for students and early-career researchers. This financial support was fundamental to broadening the impact of the event by promoting the assistance of underrepresented groups. This Meeting Review highlights the outcomes of our workshop and shows the advances of the Argentinian scientific community involved in EV research.

## Teaser

**Summary:** This Meeting Review article showcases the latest workshop of the Argentine Group for Extracellular Vesicles, emphasizing its impact in fostering national and international collaborations.

## Introduction

**Fig. 1.**
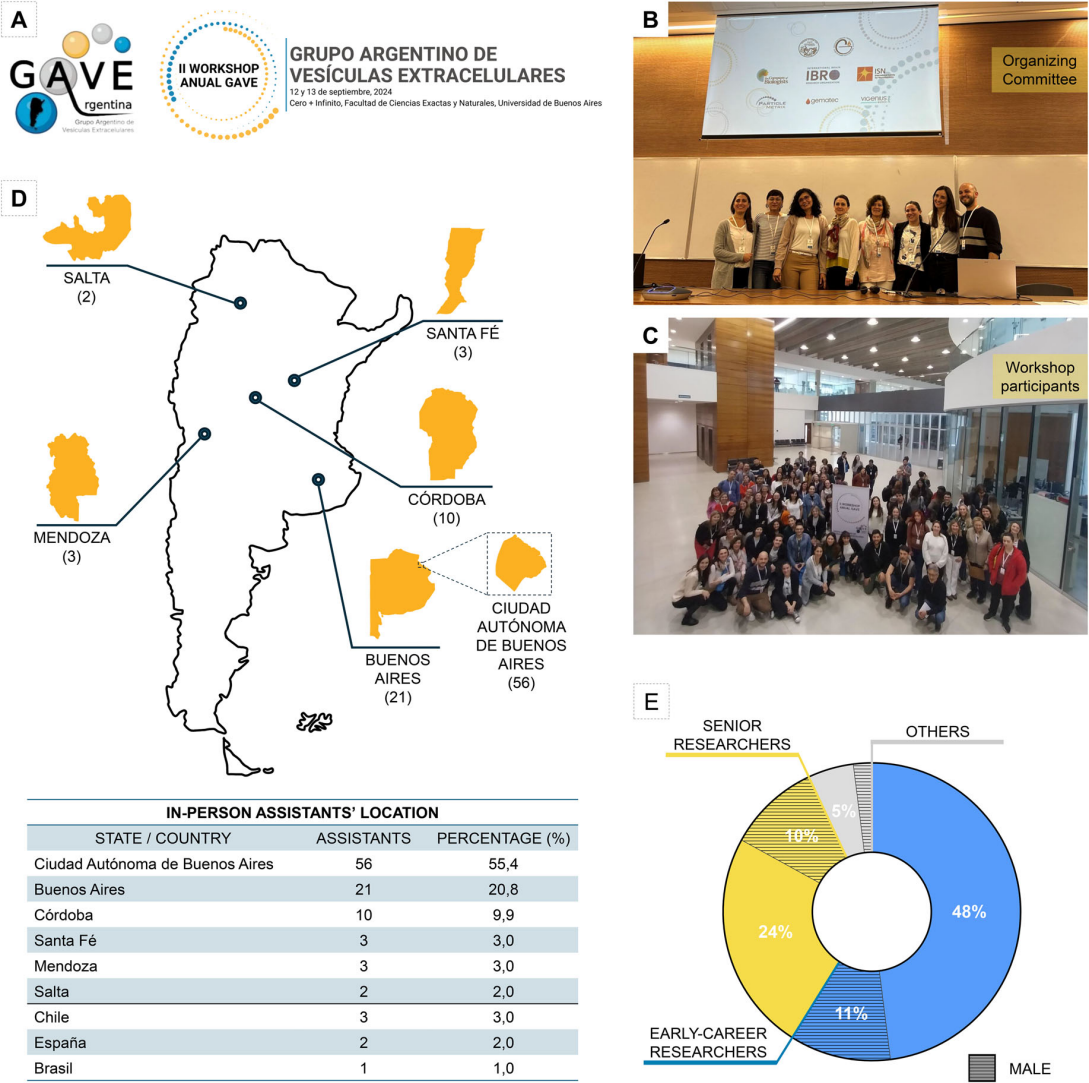
**II GAVE Workshop organization and attendee information.** (A) GAVE logo (left) and II GAVE Workshop logo (right). (B) Members of the organizing committee. (C) In-person assistants. (D) Scheme of the geographical distribution of Argentine attendees (top) and table showing the in-person assistants' locations (bottom). (E) Attendees by career stage and gender. Blue, early-career researchers; yellow, senior researchers; gray, others; line pattern, self-identifying male attendees.

Extracellular vesicles (EVs) are membranous particles released by nearly all cell types, among a wide range of prokaryotic and eukaryotic organisms. Their active role in intercellular communication, along with their ability to reflect the physiological and pathological state of their cell of origin, has triggered great interest within the scientific community worldwide (Driedonks et al., 2024). In Argentina, the Argentine Group for Extracellular Vesicles (GAVE; deriving from its Spanish name, Grupo Argentino de Vesículas Extracelulares; Fig. 1A) was created in 2022 with the main objective of gathering researchers dedicated to the study of EVs and to form a community that promotes scientific exchange in this emerging research field. GAVE comprises scientists from different regions of Argentina who investigate EVs across various fields and biological models.

Since its beginning, GAVE has aimed to foster collaboration between national and international research groups, facilitating knowledge exchange within a rapidly evolving field that continuously updates its technical tools and methodologies (Welsh et al., 2024). This has led to the formation of a group that focuses on EVs in a wide spectrum of research topics, from the EV-mediated effects of *Porphyromonas gingivalis* in pregnancy (Lara et al., 2023) to the neural crest-placode communication mediated by EV-associated microRNAs (Bernardi et al., 2024). Moreover, GAVE has been created as an inclusive and empowering space for early-career researchers and minorities to present their work.

As it is publicly known, the Argentine scientific and academic community is currently facing significant challenges, including severe financial constraints and limited government support (Orfila, 2024). Despite these obstacles, the II GAVE Workshop (Fig. 1A) successfully met over 140 assistants on 12-13 September 2024, at the Universidad de Buenos Aires (UBA), Ciudad Autónoma de Buenos Aires (CABA). The event featured three main Symposia and Plenary Lectures, including presentations by more than 13 national and international speakers who covered representative topics of the participants' primary research areas. Additionally, the workshop included oral communication and poster sessions in which over 40 early-career researchers, postdoctoral fellows, and PhD students participated.

In addition to the international (The Company of Biologists, International Society for Neurochemistry, International Brain Research Organization and Particle Metrix GmbH) and national (Gematec S.R.L. and Vigenius Biotech S.A.) financial support, the event was also supported by the Facultad de Ciencias Exactas y Naturales and the Facultad de Farmacia y Bioquímica of the UBA. This meeting was organized with the collaboration of Pamela Valva PhD, Ana Lis Moyano PhD, Marcela Brocco PhD, Carolina Jancic PhD, Ana Carolina Donadio PhD, Melisa Monteleone PhD, Vanesa Hauk PhD, and Gonzalo Guendulain PhD student, (Fig. 1B), all investigators from the Consejo Nacional de Investigaciones Científicas y Técnicas (CONICET), who come from diverse regions of Argentina and study various topics related to EVs.

## The II GAVE Workshop

The I GAVE Workshop was held in 2023, and, as our first meeting, it was organized with the idea of bringing together local researchers. Apart from gathering the EV Argentine research community, the objective of the 2024 GAVE Workshop was to reach speakers and participants from other countries. The funding received for the 2024 GAVE Workshop enabled the organization to invite international speakers and to provide financial assistance to national and international early-career researchers.

### Geographical distribution of the II GAVE Workshop participants

Since GAVE is a young community, the organizing committee conducted online surveys to gather input on key aspects such as accessible locations and dates, priority topics, preferred presentation formats, and other considerations. Based on this feedback, CABA was chosen as the workshop venue due to its better lodging options and connections with other provinces. With the support of the UBA, the meeting was held at the Cero+Infinito, a cutting-edge and technological building planned and built to promote Science, Technology, Engineering and Mathematics (STEM) higher education in national universities. UBA also generously provided all its technological facilities, enabling the II GAVE Workshop to be conducted in a hybrid format. Thus, the event hosted over 100 researchers in person (Fig. 1C), with almost 40 participants attending virtually.

Regarding the participation and federal representation within this event, the workshop joined researchers from five provinces of Argentina, with a strong presence of in-person participants from Buenos Aires and Córdoba (Fig. 1D). Despite the need to achieve a more federal representation in our following events, this geographical distribution is common in meetings held in Buenos Aires, where most of the CONICET research institutes are located. In the future, GAVE aims to increase the number of participants from other Argentine provinces, thereby enhancing federal representation in this field.

To promote the federal representation during the event, the organizing committee decided to allocate significant funds for registration fee waivers, travel grants and caregiver fellowships. Due to the selection criteria, the in-person participation of early-career researchers (undergraduate, doctoral and postdoctoral students, and Junior Researchers from CONICET) reached almost 60% of the attendees. Over 30% of the participants were senior investigators (Associate, Independent and Principal Researchers from CONICET), and the remaining were other professionals dedicated to science (laboratory managers, scientific support staff, medical doctors, among others). From our surveys, we also found that over 75% of the assistants identified as women and 25% as men (Fig. 1E).

In the 2024 GAVE meeting, we had the opportunity to invite speakers and participants from other countries in Latin America (Chile and Brazil) and Europe (Spain), therefore reaching one of the milestones of the II GAVE Workshop (Fig. 1D). Our long-term goal is to keep expanding our frontiers, including other groups interested in the EV field.

### Distribution of main research topics of the II GAVE Workshop

**Fig. 2.**
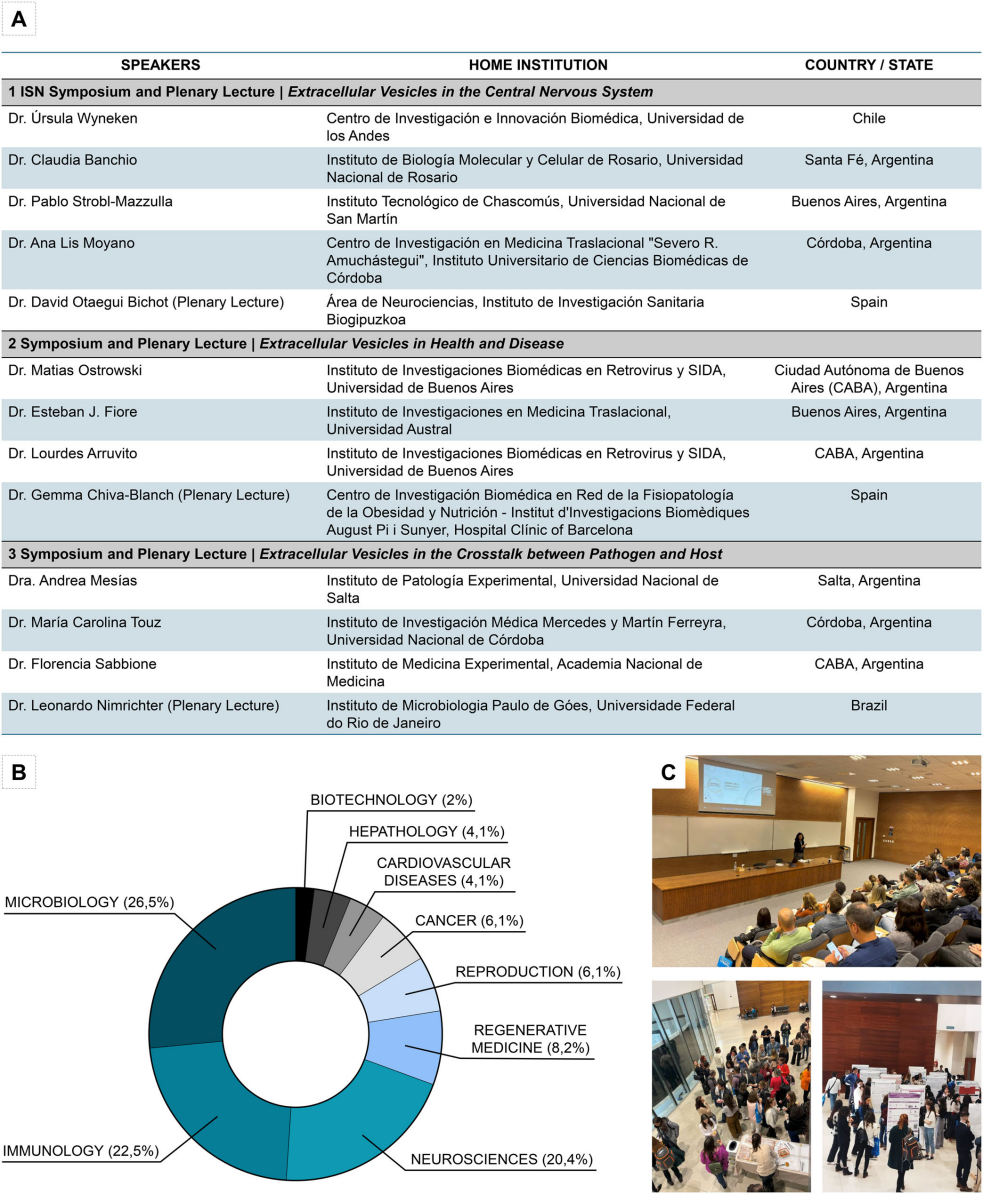
**II GAVE Workshop speakers and topic distribution.** (A) Table showing the Symposia and Plenary Lectures with their respective participants, home institution and country. (B) Topic distribution of posters and oral communications. (C) Images of the auditorium, coffee break and poster session during the event.

The main topics were selected from the pre-workshop online surveys and mainly covered EV research in neuroscience, immunology, cancer and microbiology. These topics were explored in three Symposia and Plenary Lectures that included ‘EVs in the Central Nervous System’ (neuroscience), ‘EVs in Health and Disease’ (immunology) and ‘EVs in the Crosstalk between Pathogen and Host’ (microbiology). Speakers were chosen to balance gender and represent diverse regions, including international speakers from Chile, Spain and Brazil (Fig. 2A). All of them are recognized international experts in the field eager to establish collaborations with GAVE members. Moreover, eight oral communications from PhD students, postdocs and early-career researchers were held during the event, as well as two commercial talks from our sponsors (Particle Metrix GmbH and Gematec S.R.L.). In addition to the main topics, poster sessions included EVs in regenerative medicine, reproduction, cancer, cardiovascular diseases, hepatology and biotechnology (Fig. 2B).

GAVE has been created as an inclusive and empowering space for graduate students and early-career researchers to present their work. To recognize their contributions and promote their careers, we implemented a series of prizes directed to three different categories: (1) best undergraduate poster presentation, (2) best doctoral/postdoctoral poster presentation, and (3) best doctoral/postdoctoral oral communication. These prizes were awarded to María Victoria Bühler (Buenos Aires, neurosciences), Mercyleidi Díaz Reyes (Santa Fé, neurosciences) and Brenda Lara (CABA, microbiology), respectively. For the poster sessions, highly renowned national and international researchers generously agreed to participate in the evaluation process. The main criteria assessed were the students' clarity in explaining their objectives and discoveries, as well as the poster's graphic organization and adherence to the presentation guidelines. This provided an opportunity to discuss their scientific findings and to exchange ideas with experts in their field.

Given these results, the II GAVE Workshop successfully included the main representative topics of the participants' primary research areas in the EV field in Argentina. Post-workshop online surveys confirmed this outcome, with most attendees highly satisfied by the results of the event and agreeing about the scientific excellence of the speakers and works shown during the sessions. Among the 93 attendees who responded to the survey, nearly 96% rated the topics as either ‘very interesting’ or ‘interesting’.

A special focus was also given to gender perception and equality, finally addressing the key topics of inclusivity considered important within GAVE. In the pre-registration survey, we asked questions regarding gender perception, disabilities, and care requirements for children and adults. Based on this information, we allocated funds to support caregiver fellowships, provided assistive equipment for individuals with disabilities and included participants' self-identified pronouns on their credentials, aiming to foster the most inclusive environment possible. Finally, we promoted gender equity in symposia and oral communications, achieving at least 50% female participation in each segment. In the words of the participants: “Attending the workshop and discussing science was like being in an oasis in a context of extreme uncertainty and instability”.

## Conclusions and challenges

GAVE is a recently founded scientific group dedicated to promoting EV research in Argentina. Beyond bringing together scientists from different provinces and fostering national collaborations, the 2024 II GAVE Workshop aimed to expand our local frontiers and promote both regional and international collaborations. Additionally, the workshop focused on creating a safe space for early-career researchers and underrepresented groups to actively participate and showcase their scientific work.

Historically, the Argentine scientific system has been supported and organized by national research agencies, with most research activities concentrated in universities. Consequently, most of the community and attendees of the II GAVE Workshop are affiliated with CONICET research units. According to CONICET's website, out of 351 registered academic units in Argentina, nearly 170 (48%) are in CABA and the Buenos Aires province, followed by Córdoba (45 units) and Santa Fé (31 units) (https://red.conicet.gov.ar/nomina-y-mapa-institucional/). This might explain in part the geographical distribution of the II Workshop attendees, representing nearly 90% of those locations (Fig. 1D).

As highlighted throughout this Meeting Review, during the past year, the Argentine scientific budget has been significantly reduced, with most funds not even allocated. Traditionally, this financial support helped to cover the costs of attending scientific events, and the lack of funds might have contributed to the underrepresentation from other provinces. However, we do not yet have comprehensive statistics on the national representation of EV research in Argentina. GAVE is currently evaluating the data collection with the goal of eventually conducting an integral analysis of these factors, including the geographic distribution of research groups working on EVs.

Beyond national attendees, this year's workshop featured distinguished international speakers from Latin America and Europe. While both GAVE workshops have been recognized in post-event surveys for scientific excellence, the involvement of highly renowned international scientists provides opportunities to expand our impact. In addition to promoting regional and international collaborations, this interaction offered early-career researchers a unique opportunity to share their discoveries with experts who can positively impact their work.

Special efforts were made to promote the participation of students and early-career researchers. The organizing committee allocated a significant portion of the grants to encourage broad national attendance and ensure the involvement of younger members of GAVE. As a result, registration was free for most attendees, and travel and caregiver fellowships were awarded to 100% of early-career researchers who requested them (ten travel and over ten caregiver fellowships). This initiative not only contributed to the 60% participation rate of early-career researchers but may have also played a pivotal role in the remarkable female representation (75%) during the event (Fig. 1E). As a young group, GAVE is deeply committed to promoting equity and values the enrichment that diversity brings. These efforts have undoubtedly enhanced the outcomes of our workshop, and it has been both inspiring and refreshing to create a safe space for sharing research in these challenging times for our scientific community.

Finally, the II GAVE Workshop showcased a strong representation of research on topics related to human health (immunology, neurosciences, regenerative medicine, reproduction, cancer, cardiovascular diseases and hepatology) and microbiology. A significant challenge for our community will be to engage EV researchers from other fields and biological systems, such as plant biology. In addition to our annual workshops and our social media communication activities, we strongly believe that this Meeting Review will help us reach other national and Latin American research groups, thereby diversifying and enriching our group.

While we celebrate the significant achievements made possible through the organizing committee's dedication and perseverance, the lack of long-term government support and scientific policies poses a serious threat to our globally recognized scientific system. The foundations of our community depend deeply on national political and social support. We hope that this Meeting Review serves as proof of the importance of sustained investment in a national framework and funding agencies that allow the training of new generations of scientists and drive technological development in our country.

*Hay quienes creen que la investigación científica es un lujo o un entretenimiento interesante, pero dispensable […] La disyuntiva es clara: o bien se cultiva la ciencia, la técnica y la investigación y el país es próspero, poderoso y adelanta; o bien no se practica debidamente y el país se estanca y retrocede, vive en la pobreza y la mediocridad […] La ciencia no es cara, cara es la ignorancia.*

*There are those who believe that scientific research is a luxury or an interesting but dispensable pastime […] The dilemma is clear: either science, technology, and research are nurtured, and the country becomes prosperous, powerful, and advances; or they are neglected, and the country stagnates and regresses, living in poverty and mediocrity [...] Science is not expensive, ignorance is.*

Bernardo Alberto Houssay

1947 Argentine Nobel Prize in Medicine
